# Supplementary material for: Computational analysis of US congressional speeches reveals a shift from evidence to intuition
Source: Nat Hum Behav. 2025 Apr 10;9(6):1122–33. doi: 10.1038/s41562-025-02136-2 (PMC12185346; doi:10.1038/s41562-025-02136-2)
Supplement: Supplementary file 1 — Supplementary Notes 1–12, Figs. 1–15 and Tables 1–6. [file 41562_2025_2136_MOESM1_ESM.pdf]

nature human behaviour

Supplementary information https://doi.org/10.1038/s41562-025-02136-2

Computational analysis of US congressional speeches reveals a shift from evidence to intuition

In the format provided by the authors and unedited

S1 Number of speeches

Supplementary Figure S1 shows the number of speeches per congressional session in both the House and Senate from 1879 to 2022. While there is some variation in the number of speeches across sessions, each session contains a minimum of 35,000 speeches. This provides us with a seizable amount of data for reliable analyses.

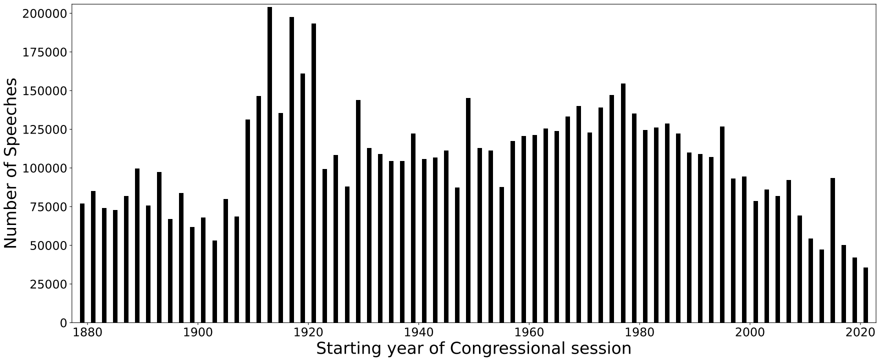

Supplementary Figure S1 Number of speeches across both House and Senate for Congressional sessions between 1879 and 2022

S2 Sample of speeches based on EMI score

Supplementary Table S1 and Supplementary Table S2 show examples of speeches with positive and negative EMI score. Supplementary Table S3 shows examples of speeches with low EMI score (in the bottom 1%) in periods with overall low EMI score in the main text (Figure 1A).

Supplementary Table S1 Examples of speeches with positive EMI score

Sample speeches
"Lives have been directly affected- by the tragedy of suicide were also called upon to provide insight into what might be done in the family and in the community to prevent the further senseless waste of young lives. By all accounts, the conference was a tremendous success. In fact, many participants returned to their communities and, with the knowledge obtained from the conference, established suicide prevention programs. To assist other communities, the Youth Suicide National Center, in conjunction with the Office of Human Services, Administration for Children, Youth, and Families, of the Department of Health and Human Services, has compiled for dissemination the findings and recommendations of the conference. I note that the findings and recommendations will be published within 1 year of the conference, thereby recognizing the urgency associated with the problem. In sum, the administration has been involved in an effort to address the tragedy of youth suicide"
"Yes. The Senator can get them in detail I am sure from the report of the Federal Trade investigation. Before I conclude I shall give some figures as to some of the holding companies and subsidiaries, and some figures applying to all of them showing the fictitious capital stocks and bonds which have been floated and sold to an innocent public, for which absolutely no real value existed."
"If the distinguished and honorable Senator has not read the report of the committee, which was prepared for the information of Senators, he must not charge the committee with any dereliction of duty in not supplying him with information."
"Is it not true that the only basis of valuation that can be established for a fixed rate of return is through the property investment account until the actual value is ascertained by the physical valuation of the railroads by the Interstate Commerce Commission?"
"May I ask the Senator from Iowa to repeat the form in which the comparison has been tabulated? My attention was diverted to another quarter at the moment. In what shape will the comparative table be?"

Supplementary Table S2 Examples of speeches with negative EMI score

Sample speeches
“I can give the Senator an illustration. I had some ancestors who were very smart people. but fought for the Stuarts in Great Britain against Puritanism and the Commonwealth and the Parliament. They were wise men individually. but historically they were asses. Does the Senator understand the illustration? Their successors partially in my person have confessed that they were asses.”
“I desire to say to the gentleman from Wisconsin. who never ”shakes his head wisely.” that he has no business to shake it unwisely at me.”
“Oh. yes. your howl about the farmers of the country and the destruction of the price of wheat is nothing but the wail of the old standpatter. who sees the mountain of protection giving way under ceaseless and constant hammering on the part of the people.”
“Mr. Speaker. I join with my colleagues in expressing my sorrow at the passing of our former colleague. the Honorable Charles A. Halleck of Indiana. It was my privilege to serve with Charlie for quite a long time. The leader on the Republican side. he was a tireless worker. candid. honest. and able. He contributed greatly to his country and to the Congress itself. May I say that his work here will be long remembered and his contributions to his district. State. and Nation will be lasting.”
“Mr. Speaker, in the face of this impasse and in the spirit of the season. I believe we should forget this Democrat versus Republican stuff, legislators versus Executive Branch, liberals versus conservatives, and unite under the common bond of being Americans. We are reminded of a similar impasse in our history at the constitutional convention when the sage elder statesman, Ben Franklin, stood with these words: ”In the beginning of our war with Britain. we prayed daily for guidance. Our prayers were heard and answered. Have we now forgotten this powerful ally? The longer I live, this I know to be true, God governs the affairs of men, for if a sparrow cannot fall without His notice, is it probable that a nation can rise without His aid? The psalmist tells us in chapter 118. verse 8. ”Put your trust in God. not confidence in men.” We have these same words above the Speakers chair and right over the American flag. I believe that we, as a Congress, should come together as Democrats and Republicans and leaders to do what is best for the American country, put God and country first, not partisan, politics.”

Supplementary Table S3 Example of speeches with low EMI score in specific periods. There is a tendency to focus on prevailing crisis, including the war in the Philippines in the 1890s, the impact of the Great Depression in the 1930s, and challenges associated with raising the debt ceiling in the 2020s.

Period	Sample speeches
1899	“Philippine policy whatever that may be right or wrong. is the veriest rot. an insult to intelligence. a shame upon manhood. a tale told by an idiot. a betrayal of the principle of self government. I am willing to go as far as anyone in patriotism. I will support the country in any emergency. but President McKinley is not the country. The time has not yet come I pray Almighty God that it may never arise when the American people will accept the arrogant dictum of Louis XIV. when repeated by an American President. ”I am the State!” If President McKinley is at all worthy of his high position. he must entertain a supreme contempt for those political invertebrates. particularly for those. claiming to be Democrats. who. in order to catch the crumbs falling from their masters table [applause]. go about saying. ”The President is wrong in his Philippine policy. but we must support”
1933	“expression of such noble sentiments I find myself utterly unable to reconcile his refusal to relieve the misery. the fears. the want. the privation. the bitter humiliation of millions of our finest men and women and children of this land. To me it is an utterly incomprehensible reversal of everything Franklin Delano Roosevelt has voiced in the past. everything he has claimed to stand for. everything he has typified to a Nation whose citizens today still stand on the perilous edge of a yawning precipice. into the darkness and social chaos of which a thrust. the like of that which the President has made into the hearts and the hopes of millions of Americans. might easily plunge us. I want to say to you. Mr. Chairman. that in the person of Franklin Delano Roosevelt. into his keeping. because of the high spiritual ideals he has voiced. the high spiritual promises he”
2021	“aside differences and move it forward. But now, unfortunately, some of our Republican colleagues even though they were eager to have Democrats support them when President Trump was President now some of our Republican colleagues are reportedly contemplating a reckless idea, spearheaded by the Republican Senator from Wisconsin, to oppose any effort to raise the debt ceiling whatsoever. And, unfortunately and sadly, the Republican leader seems to be going along. Let me be clear: taking the debt hostage and playing games with the full faith and credit of the United States is reckless, irresponsible, and will harm every single American. It is a complete non-starter. This is not just another political debate. It is about honoring our unbroken commitment to pay our debts and avoid another financial crisis at a crucial moment for our country. Now it is important to remember that this is not about green-lighting future spending”

S3 Evidence and intuition scores

Supplementary Figure S2 illustrates the trends for the evidence and intuition scores from which the EMI score is derived.

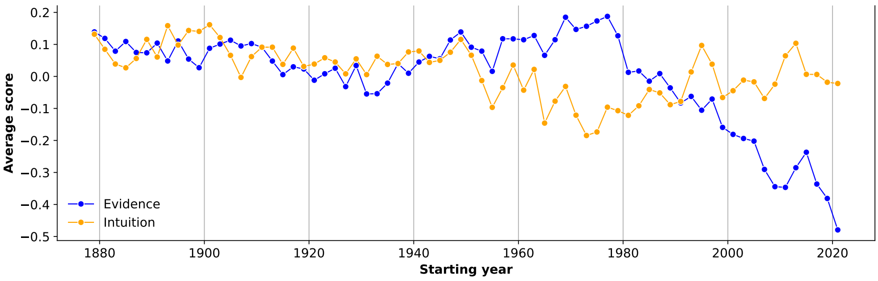

Supplementary Figure S2 The trend for the evidence and intuition scores

S4 EMI trends in the chambers of U.S. Congress

Supplementary Figure S3 shows the trend of EMI by party in both chambers of the U.S. Congress over time. The trends follow a similar pattern to the one observed for the overall EMI score in the main text.

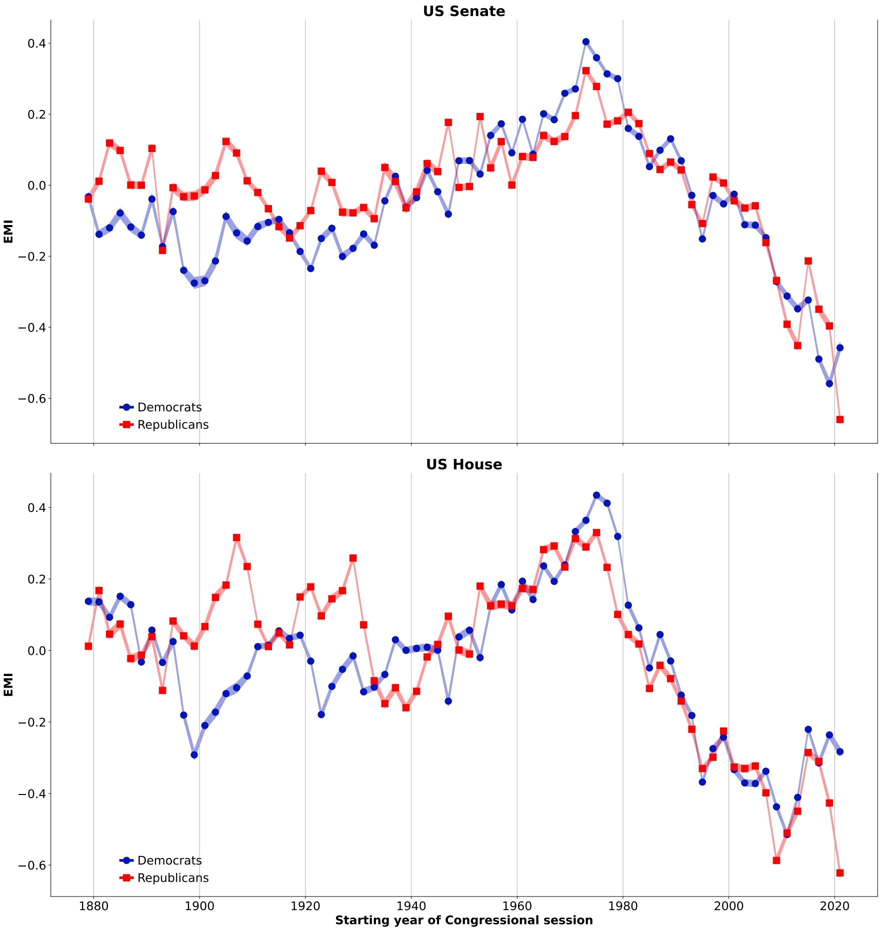

Supplementary Figure S3 Time series of EMI by party in the U.S. Senate and House

S5 Negative trend of EMI from the mid-1970s

Supplementary Figure S4 shows the fit for the linear regression model confirming the downward trend of the EMI from the legislative session with peak EMI value, 1975-76.

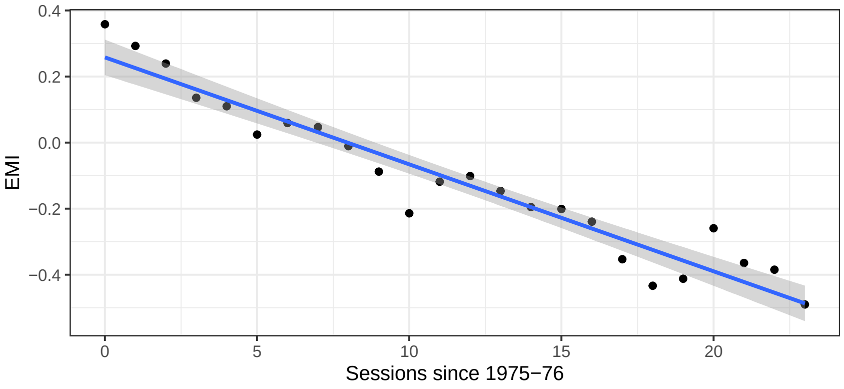

Supplementary Figure S4 EMI values after the peak in 1975-76. The line shows a linear fit and shaded area shows the standard error of the predicted EMI values based on the regression model.

S6 Scatter plot of polarization and EMI

Supplementary Figure S5 depicts the relationship between EMI and polarization.

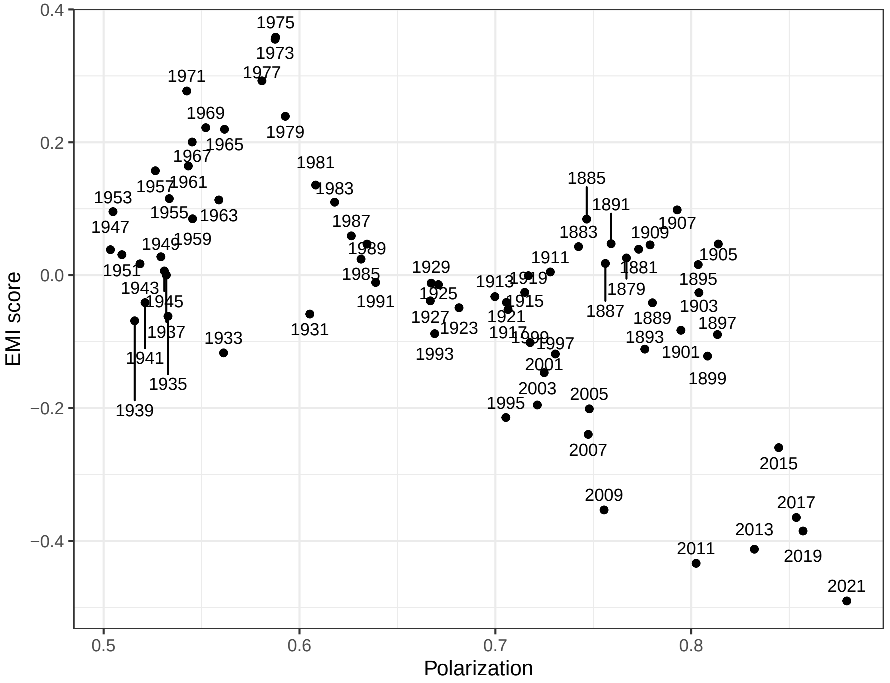

Supplementary Figure S5 Scatter plot of polarization and EMI in the same year. The Pearson's correlation coefficient between these two variables is -0.615 (95% CI = $[-0.741, -0.447]$, $p = 9.08e-09$, $N = 72$). Statistical significance was assessed using a two-sided t-test. No adjustments for multiple comparisons were made. Note that vertical lines are included solely for labeling purposes to connect the year labels to their respective points.

S7 Lagged correlation plots for EMI, polarization and inequality

Supplementary Figure S6 shows the lagged correlation for EMI and polarization, EMI and inequality, as well as polarization and inequality.

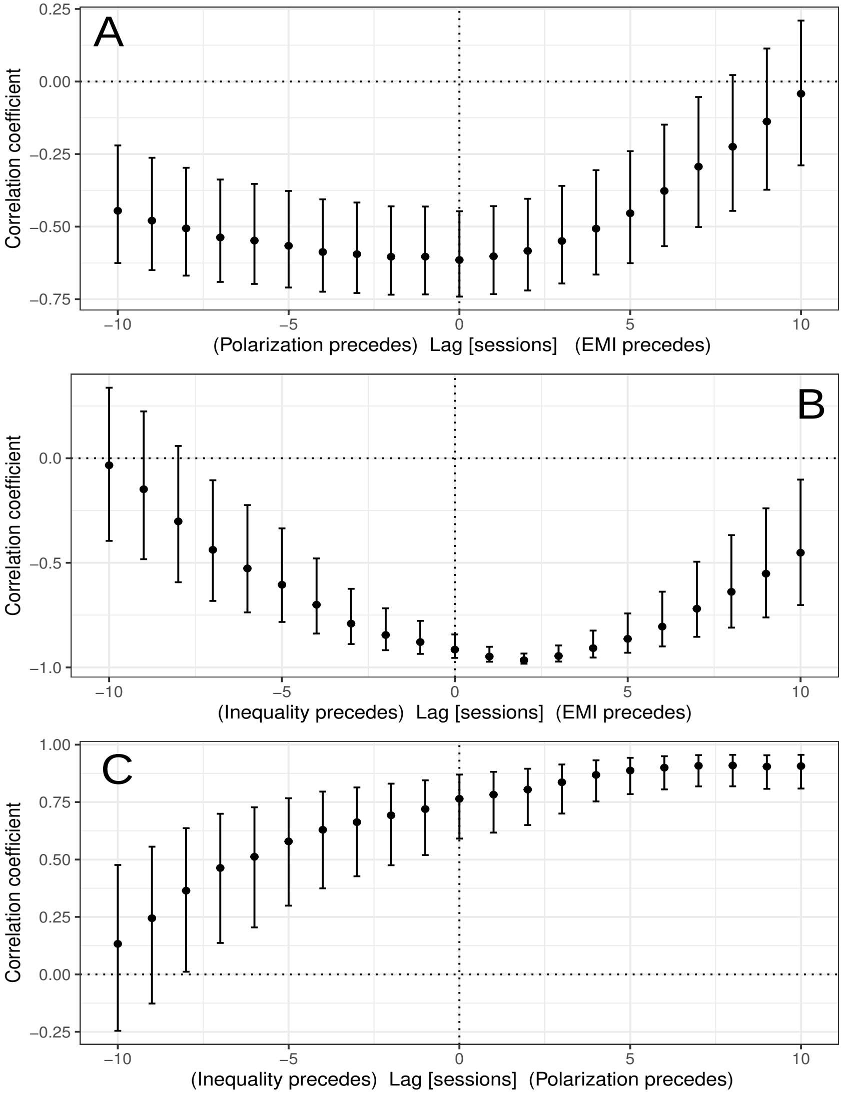

Supplementary Figure S6 Lagged correlation analysis between EMI and Polarization (A), EMI and Inequality (B) and Inequality and Polarization (C). Data are presented as Pearson's correlation coefficients, with 95% confidence intervals shown as error bands ($N = 72$).

S8 Robustness analysis of the relationship between EMI and inequality

Supplementary Table S4 shows the results, where the first model uses the Gini index as a measure of inequality. The second model uses the share of income of the top 1% of the population but includes less reliable data since 1912. The third model considers a longer lag for polarization. Supplementary Figure S7 depicts the relationship between EMI and inequality for the different model specifications at different levels of polarization. All models demonstrate that the negative relationship between EMI and inequality is robust to the level of polarization.

Supplementary Table S4 Regression results for alternative specifications of our inequality analysis. Model 1 uses the Gini index as a measure of inequality, Model 2 uses all data on the share of income of the 1% since 1912, and Model 3 uses a longer lag of 8 legislative sessions for polarization. Values in square brackets represent 95% confidence intervals. Statistical significance was assessed using a two-sided t-test. No adjustments for multiple comparisons were made.

	Ineq (Gini)<br>(1)	Ineq (since 1912)<br>(2)	Ineq<br>(3)
EMI(t-1)	**-0.24**<br>[-0.34,-0.15]<br>p=1.23e-05	**-0.22**<br>[-0.32,-0.12]<br>p=5.62e-05	**-0.14**<br>[-0.23,-0.05]<br>p=3.62e-03
Pol(t-1)	**0.09**<br>[0.02,0.16]<br>p=9.69e-03	0.02<br>[-0.05,0.09]<br>p=5.27e-01	
Gini(t-1)	**0.46**<br>[0.26,0.65]<br>p=3.53e-05		
EMI(t-1)∗Pol(t-1)	**0.25**<br>[0.12,0.37]<br>p=3.50e-04		
Ineq(t-1)		**0.70**<br>[0.51,0.89]<br>p=2.13e-09	**0.52**<br>[0.32,0.72]<br>p=8.88e-06
Pol(t-1)∗EMI(t-1)		**0.26**<br>[0.11,0.41]<br>p=8.16e-04	
Pol(t-8)			0.04<br>[-0.03,0.12]<br>p=2.15e-01
EMI(t-1)∗Pol(t-8)			0.14<br>[-0.02,0.29]<br>p=7.85e-02
Intercept	0.22<br>[0.13,0.30]<br>p=7.32e-06	0.04<br>[-0.01,0.08]<br>p=1.67e-01	0.04<br>[-0.01,0.10]<br>p=2.96e-02
Observations	38	54	31
R^2	0.98	0.87	0.97
Adjusted R^2	0.97	0.86	0.96
F Statistic	315.53<br>p=8.61e-26	81.45<br>p=5.01e-21	195.75<br>p=5.30e-19

Note: Significant coefficients at the 0.05 level are in bold.

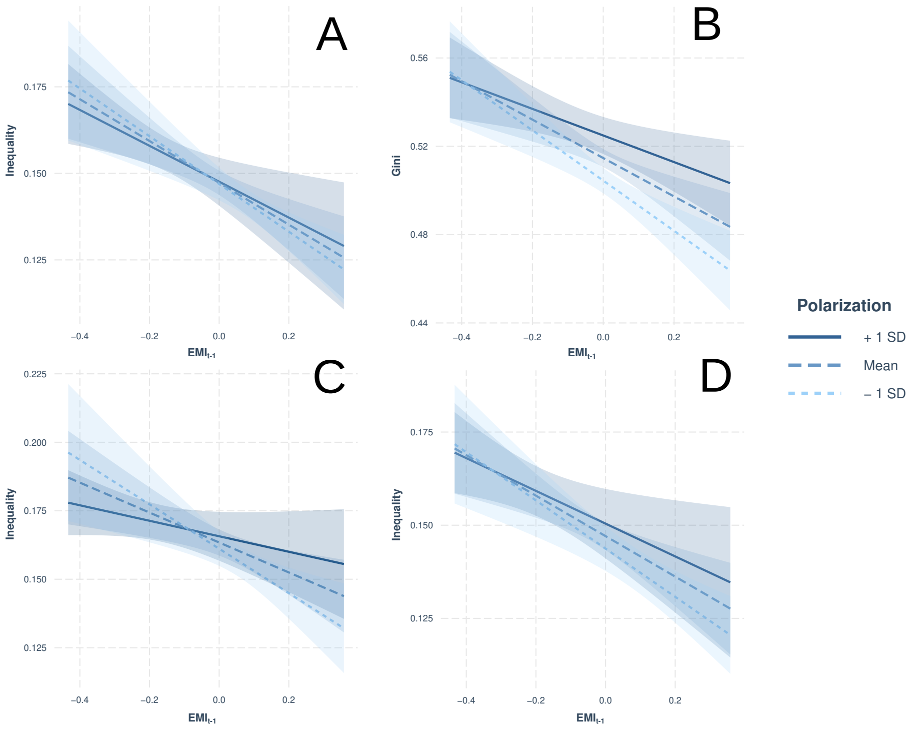

Supplementary Figure S7 Interaction plots of models of Inequality as a function of lagged values of inequality, polarization, and EMI. The figure focuses on the relationship between inequality and EMI in the previous session for three levels of polarization in the previous session: the mean and one standard deviation above and below. The shaded area around regression lines are standard errors. Panel A shows our first specification, panel B shows the result for a model with Gini index as a measure of inequality, panel C shows the result including less reliable inequality data since 1912, and panel D shows a version of our original model but including polarization values with a lag of 8.

S9 Robustness analysis of the relation between EMI and legislative productivity

Supplementary Table S5 shows the result of an alternative model specification for our analysis of legislative productivity covering a longer time period.

Supplementary Table S5 Models of Congressional productivity as a function of EMI and relevant covariates. Fits start in 1879 and include the logarithm of the number of patents as an approximation of public mood in support of more or less government policies. Data end in 2004 for MLI and LPI and in 2022 for the logarithm of the number of laws passed in the session. Values in square brackets represent 95% confidence intervals. Statistical significance was assessed using a two-sided t-test. No adjustments for multiple comparisons were made.

	MLI	MLI	LPI	LPI	nlaw	nlaw
MLI(t-1)	**0.67**	**0.59**				
	[0.48, 0.86]	[0.42, 0.75]				
	p=1.90e-09	p=3.05e-09				
Pol(t)	**-0.19**	**-0.20**	**-0.29**	**-0.31**	**-0.43**	**-0.41**
	[-0.33, -0.06]	[-0.34, -0.05]	[-0.51, -0.08]	[-0.53, -0.08]	[-0.66, -0.21]	[-0.68, -0.14]
	p=6.54e-03	p=7.90e-03	p=8.72e-03	p=8.18e-03	p=2.66e-04	p=3.19e-03
patents(t)	**0.28**	**0.37**	**0.18**	**0.22**	-0.10	-0.11
	[0.05, 0.51]	[0.16, 0.58]	[0.01, 0.35]	[0.05, 0.39]	[-0.26, 0.05]	[-0.35, 0.12]
	p=1.92e-02	p=8.66e-04	p=4.36e-02	p=1.27e-02	p=1.91e-01	p=3.31e-01
PartyControl(t)	0.07	0.10	**0.14**	**0.17**	0.23	0.25
	[-0.07, 0.21]	[-0.04, 0.24]	[0.01, 0.28]	[0.03, 0.30]	[-0.07, 0.54]	[-0.07, 0.58]
	p=3.23e-01	p=1.42e-01	p=4.08e-02	p=1.45e-02	p=1.27e-01	p=1.21e-01
PartyControlDif(t)	0.10	0.11	0.04	0.04	-0.25	-0.25
	[-0.06, 0.25]	[-0.04, 0.26]	[-0.10, 0.18]	[-0.10, 0.18]	[-0.58, 0.08]	[-0.58, 0.08]
	p=2.19e-01	p=1.61e-01	p=6.08e-01	p=5.42e-01	p=1.29e-01	p=1.32e-01
EMI(t)		**0.11**		0.07		0.05
		[0.01, 0.21]		[-0.03, 0.18]		[-0.16, 0.25]
		p=2.80e-02		p=1.60e-01		p=6.33e-01
EMI(t)*Pol(t)		-0.02		0.00		-0.05
		[-0.19, 0.15]		[-0.16, 0.17]		[-0.26, 0.15]
		p=8.18e-01		p=9.68e-01		p=6.08e-01
LPI(t-1)			**0.63**	**0.58**		
			[0.41, 0.84]	[0.37, 0.80]		
			p=4.26e-07	p=1.27e-06		
nlaw(t-1)					**0.33**	**0.32**
					[0.09, 0.57]	[0.08, 0.57]
					p=7.95e-03	p=1.12e-02
Intercept	-0.02	-0.06	-0.08	-0.11	-0.02	-0.06
	[-0.12, 0.08]	[-0.17, 0.05]	[-0.17, 0.01]	[-0.21, -0.02]	[-0.24, 0.20]	[-0.33, 0.20]
	p=7.20e-01	p=3.05e-01	p=8.92e-02	p=2.11e-02	p=8.47e-01	p=6.34e-01
Observations	62	62	62	62	70	70
R^2	0.94	0.94	0.94	0.95	0.63	0.63
Adjusted R^2	0.93	0.93	0.94	0.94	0.60	0.59
F statistic	167.22	125.51	186.41	133.02	21.39	14.98
	p=2.36e-32	p=4.52e-31	p=1.36e-33	p=1.04e-31	p=1.63e-12	p=2.85e-11

Note: Significant coefficients at the 0.05 level are in bold.

S10 Topic analysis

To address concerns on the impact of topic composition, we train a classifier (based on transformers [1]) on the CAP (Comparative Agendas Project) dataset covering 20 policy areas and a non-policy category, `Others` (Source: https://www.comparativeagendas.net/us). The classifier (available at: https://huggingface.co/saroyehun/CAP_classifier) predicts the most likely policy area covered by each piece of text. We report the performance of the classifier (F1 score) on the unseen test set in Supplementary Table S6. We plot the EMI score over time for each policy area (normalizing within each topic). Supplementary Figure S8 shows very similar trend across all policy areas (and a non-policy category, 'Others').

Supplementary Table S6 F1 score for each CAP topic and macro average F1 score

Topic	F1 score
Macroeconomics	0.8303
Civil rights	0.7676
Health	0.8886
Agriculture	0.8439
Labor	0.7818
Education	0.9005
Environment	0.8481
Energy	0.8629
Immigration	0.8682
Transportation	0.8731
Law and crime	0.8207
Social welfare	0.7957
Housing	0.8462
Domestic commerce	0.8421
Defense	0.8627
Technology	0.8333
Foreign trade	0.8269
International affairs	0.8907
Government operations	0.8777
Public lands	0.8758
Others	0.6543
Macro average	0.8573

To further examine the impact of topic composition on the trends that we observed, we compute the EMI score as a macro-average over the EMI score within each topic. The resulting trend in Supplementary Figure S9 aligns very closely with our initial findings, importantly capturing the shift in the mid-1970s.

Furthermore, Supplementary Figure S10 shows the aggregate EMI score for each policy area. Some policy areas reflect more evidence-based language (such as technological issues) while others (such as civil rights) tend to use more intuition-based language. Notably, speeches that do not fall into any specific policy area tend to rely more on intuition-based language.

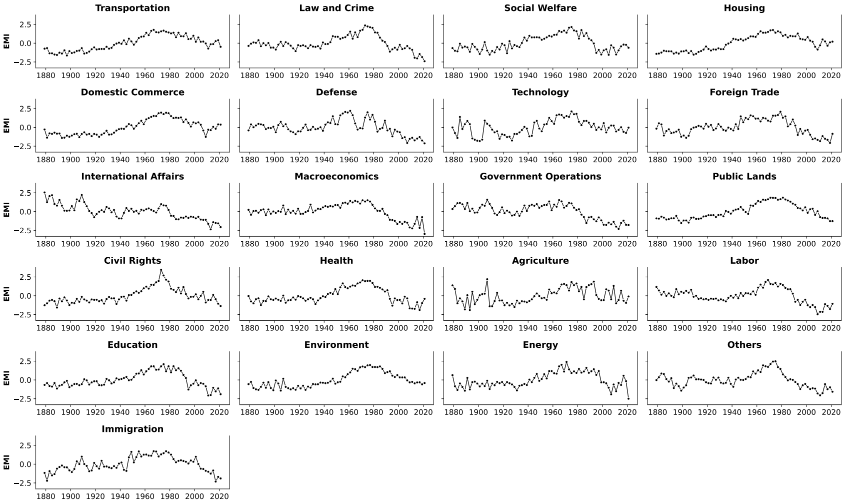

Supplementary Figure S8 EMI score over time for each topic. The EMI score follows very similar trend across all topics.

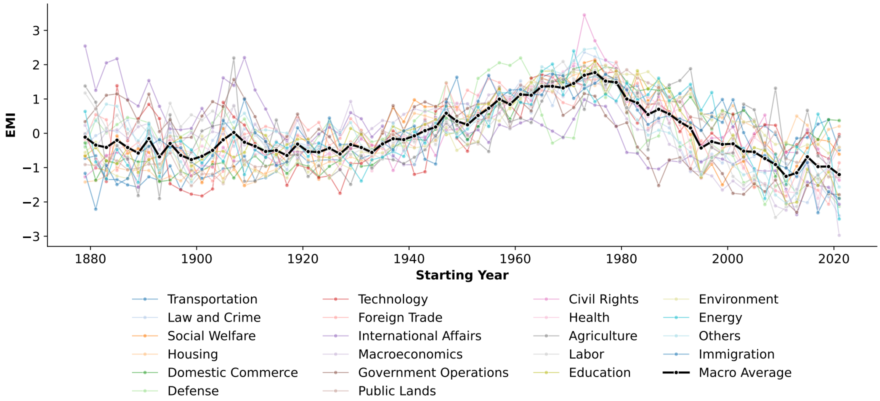

Supplementary Figure S9 EMI score for each topic corresponding to CAP policy areas (including Others to capture non-policy discourse) and the macro average over all topics

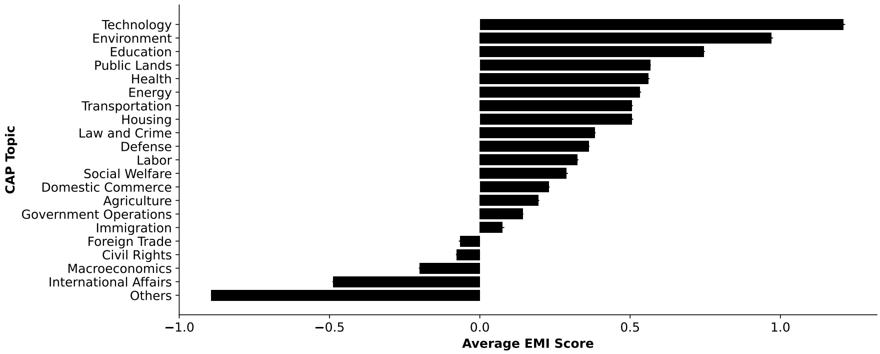

Supplementary Figure S10 Aggregate EMI score for each topic in the US Congressional speeches

S11 Analysis using temporal embeddings

To examine the potential impact of semantic change, we train temporal embeddings models on splits of the corpus into 2-decade slices. To ensure comparability, we downsample recent periods to achieve a uniform number of tokens (about 100M following [2]) across all temporal slices. We train an initial embeddings model on the combined data across slices and then fine-tune it for each 2-decade slice. This approach ensures that the temporal embeddings are aligned, allowing for comparisons over time [3, 4]. The goal of this analysis is to assess the influence of semantic change on our results. However, the measure derived from this approach is not a substitute for the main result for two key reasons: (1) the reduced data available to train the embeddings model, and (2) the vocabulary coverage of the embeddings model is smaller compared to the full corpus. Despite these, we expect the patterns observed with temporal embeddings to align closely with our main result if semantic relationships are relatively stable over time. To quantify the consistency of meaning for the keywords in our analysis, we compute the average pairwise cosine similarity between each dictionary word across all combinations of time periods (i.e., two decades) using the respective temporal embeddings. As a baseline, we calculate the average similarity of each dictionary word when paired with a randomly selected word. The results for both the evidence (Supplementary Figure S11) and intuition (Supplementary Figure S12) dictionaries show higher average similarities compared to the baseline. While there is some variation in the average similarity across time periods, it remains substantially higher than those observed in the baseline.

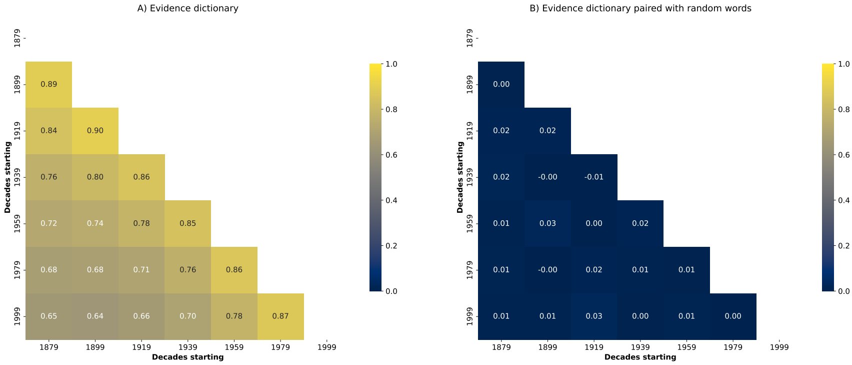

Supplementary Figure S11 Average pairwise cosine similarity for keywords in the evidence dictionary (A) and the keywords paired with random words (B)

To further support our findings, we compute the EMI score using the temporal embeddings. Specifically, we apply embeddings for a given time period to documents from the same time period and the dictionary. The results (Supplementary Figure S13) indicate that the trend of the EMI score is very similar

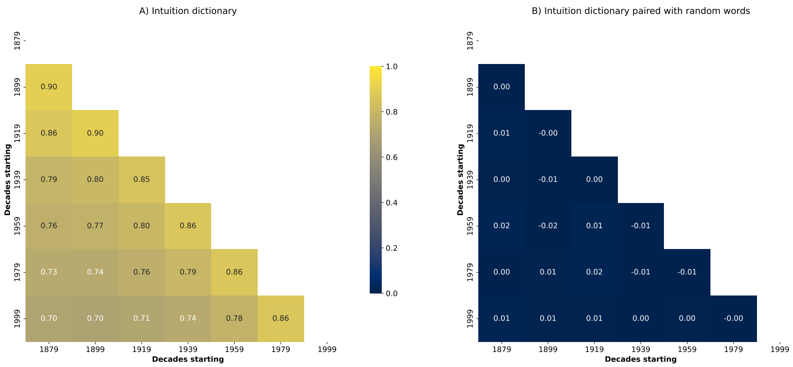

Supplementary Figure S12 Average pairwise cosine similarity for keywords in the intuition dictionary (A) and the keywords paired with random words (B)

to the one computed using an embeddings model trained on the full corpus, in particular the downward trend from the mid-1970s. Taken together, these analyses demonstrate that the impact of language change on our findings is minimal.

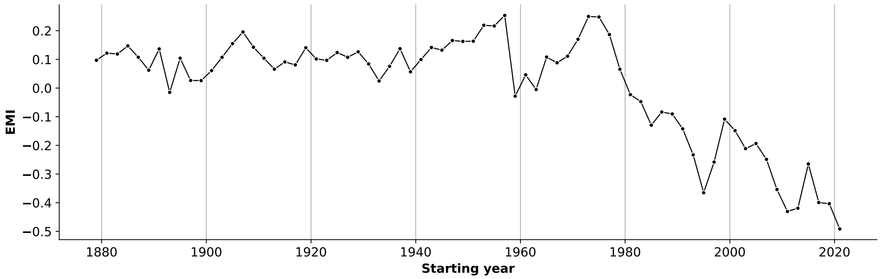

Supplementary Figure S13 Trend on the EMI score based on temporal embeddings trained on 2-decade bins and downsampled for uniform token count across time periods

S12 Identification of breakpoints

We fit a Multivariate Adaptive Regression Splines (MARS) model [5] using *py-earth* in Python. We apply this model to two versions of the EMI series: one computed using full corpus embeddings and the other using temporal embeddings (trained on 2-decade bins with downsampling). Both versions also include macro-average over topics. Supplementary Figure S14 indicates a breakpoint in the session starting in 1973, which is very close to the maximum EMI score observed in 1975. We further confirm this finding using another specification that uses the EMI scores computed without applying macro-average over topics in Supplementary Figure S15.

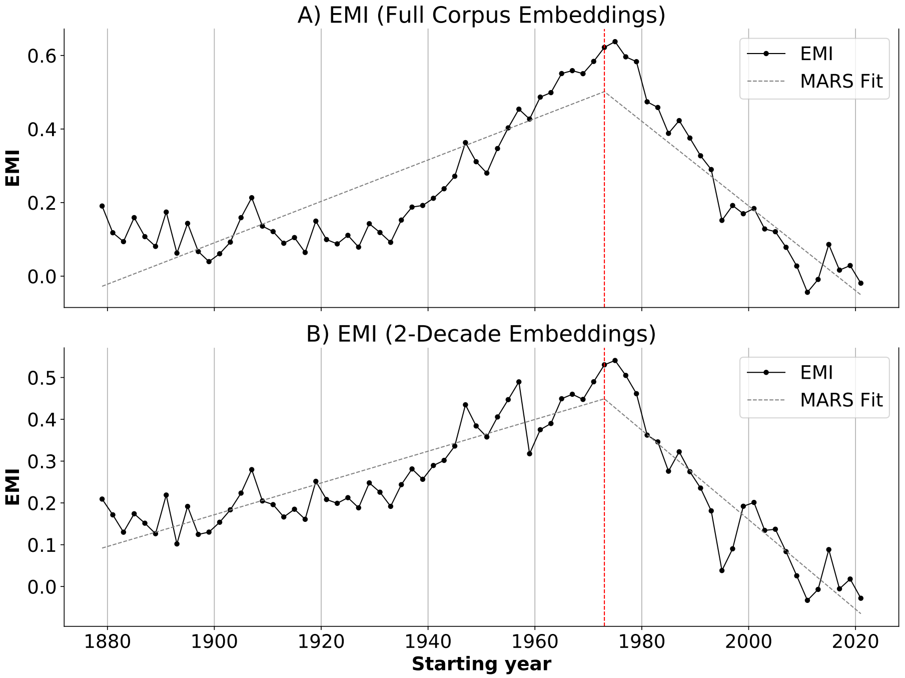

Supplementary Figure S14 Breakpoints identified using the MARS model on the EMI trend computed with full corpus embeddings (A) and temporal embeddings (B) including macro average over topics

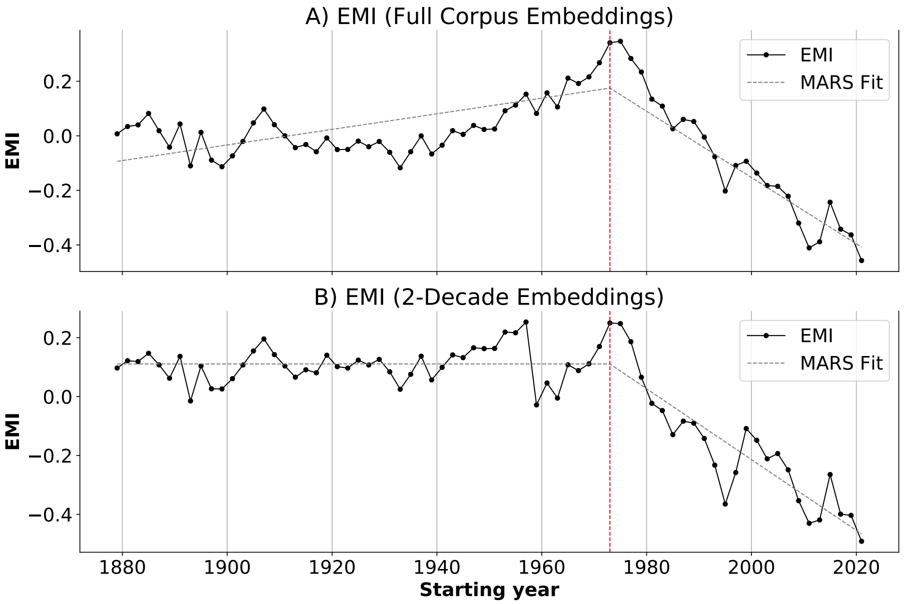

Supplementary Figure S15 Breakpoints identified using the MARS model on the EMI trend computed with full corpus embeddings (A) and temporal embeddings (B) without macro average over topics

References

[1] Devlin, J., Chang, M.-W., Lee, K., Toutanova, K.: BERT: Pre-training of deep bidirectional transformers for language understanding. In: Proceedings of the 2019 Conference of the North American Chapter of the Association for Computational Linguistics: Human Language Technologies, Volume 1 (Long and Short Papers), pp. 4171–4186. Association for Computational Linguistics, Minneapolis, Minnesota (2019). https://doi.org/10.18653/v1/N19-1423

[2] Hamilton, W.L., Leskovec, J., Jurafsky, D.: Diachronic word embeddings reveal statistical laws of semantic change. In: Proceedings of the 54th Annual Meeting of the Association for Computational Linguistics (Volume 1: Long Papers), pp. 1489–1501. Association for Computational Linguistics, Berlin, Germany (2016). https://doi.org/10.18653/v1/P16-1141

[3] Di Carlo, V., Bianchi, F., Palmonari, M.: Training temporal word embeddings with a compass. In: Proceedings of the AAAI Conference on Artificial Intelligence, vol. 33, pp. 6326–6334 (2019)

[4] Kim, Y., Chiu, Y.-I., Hanaki, K., Hegde, D., Petrov, S.: Temporal analysis of language through neural language models. In: Proceedings of the ACL 2014 Workshop on Language Technologies and Computational Social Science, pp. 61–65 (2014)

[5] Friedman, J.H.: Multivariate adaptive regression splines. The annals of statistics **19**(1), 1–67 (1991)
