# Supplementary material for: Computational analysis of US congressional speeches reveals a shift from evidence to intuition
Source: Nat Hum Behav. 2025 Apr 10;9(6):1122–33. doi: 10.1038/s41562-025-02136-2 (PMC12185346; doi:10.1038/s41562-025-02136-2)
Supplement: Supplementary file 2 — Reporting Summary [file 41562_2025_2136_MOESM2_ESM.pdf]

nature portfolio

Corresponding author(s): Stephan Lewandowsky

Last updated by author(s): February 5, 2025

Reporting Summary

Nature Portfolio wishes to improve the reproducibility of the work that we publish. This form provides structure for consistency and transparency in reporting. For further information on Nature Portfolio policies, see our Editorial Policies and the Editorial Policy Checklist.

Statistics

For all statistical analyses, confirm that the following items are present in the figure legend, table legend, main text, or Methods section.

n/a	Confirmed	
☐	☒	The exact sample size (*n*) for each experimental group/condition, given as a discrete number and unit of measurement
☐	☒	A statement on whether measurements were taken from distinct samples or whether the same sample was measured repeatedly
☐	☒	The statistical test(s) used AND whether they are one- or two-sided<br>*Only common tests should be described solely by name; describe more complex techniques in the Methods section.*
☐	☒	A description of all covariates tested
☐	☒	A description of any assumptions or corrections, such as tests of normality and adjustment for multiple comparisons
☐	☒	A full description of the statistical parameters including central tendency (e.g. means) or other basic estimates (e.g. regression coefficient) AND variation (e.g. standard deviation) or associated estimates of uncertainty (e.g. confidence intervals)
☐	☒	For null hypothesis testing, the test statistic (e.g. *F*, *t*, *r*) with confidence intervals, effect sizes, degrees of freedom and *P* value noted<br>*Give P values as exact values whenever suitable.*
☒	☐	For Bayesian analysis, information on the choice of priors and Markov chain Monte Carlo settings
☒	☐	For hierarchical and complex designs, identification of the appropriate level for tests and full reporting of outcomes
☒	☐	Estimates of effect sizes (e.g. Cohen's *d*, Pearson's *r*), indicating how they were calculated

Our web collection on statistics for biologists contains articles on many of the points above.

Software and code

Policy information about availability of computer code

Data collection	Data was collected with Python (v3.6.13) script. Specifically, the third-party Python package congressional-record (https://github.com/unitedstates/congressional-record) was used to update the existing collection of Congressional speeches.
Data analysis	Data was collected and analysed with Python (v3.6.13) and R (v4.3.1) scripts. The word embeddings models were trained using the implementation of Word2Vec algorithm in the Gensim library (v3.4.0). We efficiently apply the word embeddings using sentence-transformers library (v2.2.2). Codes for data analysis are available in a Github repository (https://github.com/saroyehun/EvidenceMinusIntuition) with a snapshot at https://doi.org/10.5281/zenodo.14288137.

For manuscripts utilizing custom algorithms or software that are central to the research but not yet described in published literature, software must be made available to editors and reviewers. We strongly encourage code deposition in a community repository (e.g. GitHub). See the Nature Portfolio guidelines for submitting code & software for further information.

Data

Policy information about availability of data

All manuscripts must include a data availability statement. This statement should provide the following information, where applicable:

- Accession codes, unique identifiers, or web links for publicly available datasets
- A description of any restrictions on data availability
- For clinical datasets or third party data, please ensure that the statement adheres to our policy

Congressional speeches are available from https://data.stanford.edu/congress_text and https://www.govinfo.gov(retrieved using https://github.com/unitedstates/congressional-record/). DW-NOMINATE scores are from https://voteview.com. Inequality data are from https://wid.world/. Data on number of patents are from https://www.uspto.gov/web/offices/ac/ido/oeip/taf/h_counts.htm. Data on public policy mood are available from https://stimson.web.unc.edu/data/. Data on legislative productivity are available from https://doi.org/10.7910/DVN/ILILUD and https://osf.io/mrghc. All the data used in this study are deposited in an Open Science Framework (OSF) repository (https://doi.org/10.17605/OSF.IO/Z6UTW).

Research involving human participants, their data, or biological material

Policy information about studies with human participants or human data. See also policy information about sex, gender (identity/presentation), and sexual orientation and race, ethnicity and racism.

Reporting on sex and gender	For the validation of dictionaries and the EMI score, participants were recruited through Prolific. For these validation tasks, we were not concerned with analysis based on sex or gender and did not directly collect this information. The validation analyses aimed solely to obtain assessments from a sample broadly representative of the U.S. population. However, information available on our sample from Prolific indicates the following:<br>- For dictionary validation, participants in our sample self-identified as 19 male, 30 female, and 1 non-binary.<br>- For EMI score validation, participants in our sample self-identified as 80 female, 72 male, 3 preferred not to say, and 1 had missing data.
Reporting on race, ethnicity, or other socially relevant groupings	We did not collect information on race, ethnicity, or other socially relevant groupings.
Population characteristics	For the validation of the computational text analysis: representative sample of adults in the United States.
Recruitment	For the validation of the computational text analysis: Prolific
Ethics oversight	The dictionary validation task was carried out under Ethics approval from the University of Bristol (ethics application #12299). For the validation of the EMI score, the University of Konstanz ethics review board granted an ethics review exemption for the annotation task used to validate the EMI score.

Note that full information on the approval of the study protocol must also be provided in the manuscript.

Field-specific reporting

Please select the one below that is the best fit for your research. If you are not sure, read the appropriate sections before making your selection.

☐ Life sciences ☒ Behavioural & social sciences ☐ Ecological, evolutionary & environmental sciences

For a reference copy of the document with all sections, see nature.com/documents/nr-reporting-summary-flat.pdf

Behavioural & social sciences study design

All studies must disclose on these points even when the disclosure is negative.

Study description	Quantitative text analysis of US Congressional Speeches from 1879 to 2022.
Research sample	The speeches of U.S. Congress Members that were analysed in this study were compiled from publicly available sources, mainly https://www.govinfo.gov/ and https://data.stanford.edu/congress_text. The dataset includes speeches from both the House of Representatives and the Senate, spanning the period from 1879 to 2022. Demographic attributes such as age and sex were not directly available for all records. However, given that the dataset encompasses all transcribed speeches from U.S. Congress members during this period, it provides a comprehensive representation of Congressional discourse over time. The selection of this dataset ensures that the analyses captures the full range of legislative rhetoric over time without potential bias introduced by sampling constraints.
Sampling strategy	The speeches included in this study are a comprehensive collection of transcribed speeches made on the floor of US Congress from 1879 to 2022. No sampling was involved in the compilation of the data and no sample size calculation was performed. Given the comprehensive nature of the dataset, its size is sufficient to support robust analyses without the need for sampling.

Data collection	Datasets were retrieved through download and automatic parsing of U.S. Congressional records. Since data collection involved no direct researcher-participant interaction, no additional individuals were present. As the study is observational and based on archival data, researcher blinding to experimental conditions and hypotheses does not apply.
Timing	The data spans Congressional sessions from January 1879 to December 2022.
Data exclusions	We excluded a total of 7,341,187 entries, including 7,336,909 duplicates and procedural speeches, as well as 4,278 noisy text (such as those resulting from Optical Character Recognition rendering of tables). We included only speeches from Democrat and Republican Congress Members.
Non-participation	There are no known cases of non-participation as this is an observational study.
Randomization	We did not perform randomization because this study is purely observational.

Reporting for specific materials, systems and methods

We require information from authors about some types of materials, experimental systems and methods used in many studies. Here, indicate whether each material, system or method listed is relevant to your study. If you are not sure if a list item applies to your research, read the appropriate section before selecting a response.

Materials & experimental systems

n/a	Involved in the study	
☒	☐	Antibodies
☒	☐	Eukaryotic cell lines
☒	☐	Palaeontology and archaeology
☒	☐	Animals and other organisms
☒	☐	Clinical data
☒	☐	Dual use research of concern
☒	☐	Plants

Methods

n/a	Involved in the study	
☒	☐	ChIP-seq
☒	☐	Flow cytometry
☒	☐	MRI-based neuroimaging

Plants

Seed stocks	N/A
Novel plant genotypes	N/A
Authentication	N/A
